# Love on wings, a Dof family protein regulates floral vasculature in *Vigna radiata*

**DOI:** 10.1186/s12870-019-2099-x

**Published:** 2019-11-14

**Authors:** Wuxiu Guo, Xue Zhang, Qincheng Peng, Da Luo, Keyuan Jiao, Shihao Su

**Affiliations:** 1 State Key Laboratory of Biocontrol and Guangdong Key Laboratory of Plant Resources, School of Life Sciences, Sun Yat-sen University, Haizhu district, Guangzhou, 510275 Guangdong China; 2 Institute of Traditional Chinese Medicine and Natural Products, College of Pharmacy, Jinan University, Guangzhou, 510632 Guangdong China; 3 Institute of Transformative Bio-Molecules (WPI-ITbM), Nagoya University, Furo-cho, Chikusa-ku, Nagoya, Aichi 464-8601 Japan

**Keywords:** Keel blossom, Landing platform, Floral vasculature, Organ asymmetry, Dof-like factor, *Vigna radiata*

## Abstract

**Background:**

The interaction among plants and their pollinators has been a major factor which enriched floral traits known as pollination syndromes and promoted the diversification of flowering plants. One of the bee-pollination syndromes in Faboideae with keel blossoms is the formation of a landing platform by wing and keel petals. However, the molecular mechanisms of elaborating a keel blossom remain unclear.

**Results:**

By performing large scale mutagenesis, we isolated and characterized a mutant in *Vigna radiata*, *love on wings* (*low*), which shows developmental defects in petal asymmetry and vasculature, leading to a failure in landing platform formation. We cloned the locus through map-based cloning together with RNA-sequencing (RNA-seq) analysis. We found that *LOW* encoded a nucleus-localized Dof-like protein and was expressed in the flower provascular and vascular tissues. A single copy of LOW was detected in legumes, in contrast with other taxa where there seems to be at least 2 copies. Thirty one Dof proteins have been identified from the *V. radiata*’s genome, which can be further divided into four Major Cluster of Orthologous Groups (MCOGs). We also showed that ectopic expression of *LOW* in Arabidopsis driven by its native promoter caused changes in petal vasculature pattern.

**Conclusions:**

To summarize, our study isolated a legume Dof-like factor LOW from *V. radiata*, which affects vasculature development in this species and this change can, in turn, impact petal development and overall morphology of keel blossom.

## Background

The majority of flowering plants have different strategies in order to attract pollinators, such as alterations in floral color, size, scent, nectar as well as shape. These changes are, in turn, under selection by different pollinators, resulting in a collection of floral traits known as pollination syndromes [1]. It has been proposed that Faboideae species with keel blossoms show adaptation towards bee pollination [2–5]. Different petals on a keel blossom play different roles in terms of pollination: the dorsal petal (or vexillum or standard or flag) acts as a billboard to attract pollinators; the ventral petals (or keel or carina) provide a space which protects the sporophyll column; and the lateral petals (or wing or alae) together with the ventral petals form a wing-keel complex, serving as a landing platform for the insects [4, 5]. Although we already know that *CYCLOIDEA*-like (*CYC*-like) genes are involved in the differentiation of petals along dorsal-ventral axis, it is still unclear how the elaborate petal shape is formed and how it leads to the genesis of a landing platform [6–8]. Organ shape and vasculature are closely linked during the evolution of flowering plants [9, 10]. The analyses of mutants with abnormal shape and vasculature in assorted lateral organs have provided new insights on the relationship between them [11, 12]. During leaf organogenesis, the final leaf form temporally coordinates with the formation of major veins, whereas the pattern of the minor veins does not completely reflect the final leaf shape [9]. Further studies unveiled complex mechanisms and genetic networks in the control of vascular tissue development, coordinated by different phytohormones, several signal peptides and multiple transcription factors [13–16]. Nevertheless, most of the conclusions are drawn from limited model species. Hence, the scenarios in other plants are still obscure, especially when referring to the origin of novel developmental traits, such as keel blossoms. The *Dof* genes encode plant-specific transcription factors, which have a highly conserved DNA-binding Dof domain [17–20]. *Dof* genes are ubiquitous in angiosperms, gymnosperms and other early diverged lineages such as moss and algae. However, the number of *Dof* genes is highly variable among green plants and tends to be proportional with morphological complexity of plant species [17, 20]. Many *Dof* genes (20 out of total 36 in *Arabidopsis thaliana*) are expressed in the vascular system, suggesting their roles during the development and function of vascular tissues [21, 22]. In Arabidopsis, different sub-clades of *Dof2.4* and *Dof5.8* are expressed in distinct early stages of leaf vasculature: *Dof2.4* is highly expressed in the primary vein of leaf primordia, while *Dof5.8* shows high expression in both primary and secondary veins, as well as petal vasculature, stamens and carpels [23, 24]. No apparent phenotype was observed in the single mutant of *dof5.8*, but it enhanced the cotyledon vascular defects of a weak allele of *auxin response factor 5–2*, indicating that Dof5.8 functions in an auxin-dependent regulation [25]. Another close paralog *Dof3.4*, or *DOF TF OBF BINDING PROTEIN 1*, which shows similar expression to *Dof5.8*, may act redundantly in the control of leaf vascular development [26]. Dof5.6 or HIGH CAMBIAL ACTIVITY2, another sub-clade of Dof transcription factors, predominantly exists in the vascular tissues of assorted organs and its gain-of-function mutant shows pleiotropic morphological changes including increased cambial activity [27]. A recent study found that cytokinin promotes the expression of a group of *Dof* genes designated as *PHLOEM EARLY DOF* in the procambial tissue, including *Dof1.1*, *Dof2.4*, *Dof3.2*, *Dof5.1*, *Dof5.3* and *Dof5.6* [28]. Multiple loss-of-function Arabidopsis Dof mutants exhibit variably reduced radial growth around early protophloem-sieve-element cells, causing further reduction of cell number in root vasculatures [28]. In this study, we evaluated a legume crop *Vigna radiata*, also known as mung bean, which is of great economic importance in Asia. Unlike classic Faboideae species with zygomorphic flowers, a part of *Vigna* spp. including *V. radiata*, have a left-handed asymmetrical flower with the left wing-keel complex generating a landing platform [29]. By large scale mutagenesis, we isolated and characterized a floral mutant *love on wings* (*low*), whose left-wing petal attaches to the ventral petal and thus, leads to a failure in landing platform formation. We observed abnormality in the petal vasculature accompanied with changes in petal shape and asymmetry. We further cloned the *LOW* locus, which encodes a plant specific Dof-like transcription factor localized to the nucleus and expressed in the flower vascular tissues. A single copy of LOW was detected in legumes in contrast with other taxa, and we found that ectopic expression of *LOW* in Arabidopsis disrupted the petal vasculature. Altogether, we infer that LOW plays an essential role in floral vascular development of keel blossoms.

## Results

### *V. radiata* has a left-handed keel blossom

The wild-type (WT) *V. radiata* flower exhibits a left-handed keel blossom (Additional file 1: Figure S1). The right lateral petal encloses the right ventral petal, whilst the left lateral petal is inflexed over the spur developed on left ventral petal, and together they form the landing platform on the left side of the flower (Additional file 1: Figure S1A). We observed that the honeybee alighted on the left landing platform and forced its head towards the base of the dorsal petal, where there is a narrow gap for the insect to insert its proboscis to the nectary (Additional file 1: Figure S1B-C).

### Characterization of the *love on wings* (*low*) mutant

**Fig. 1:**
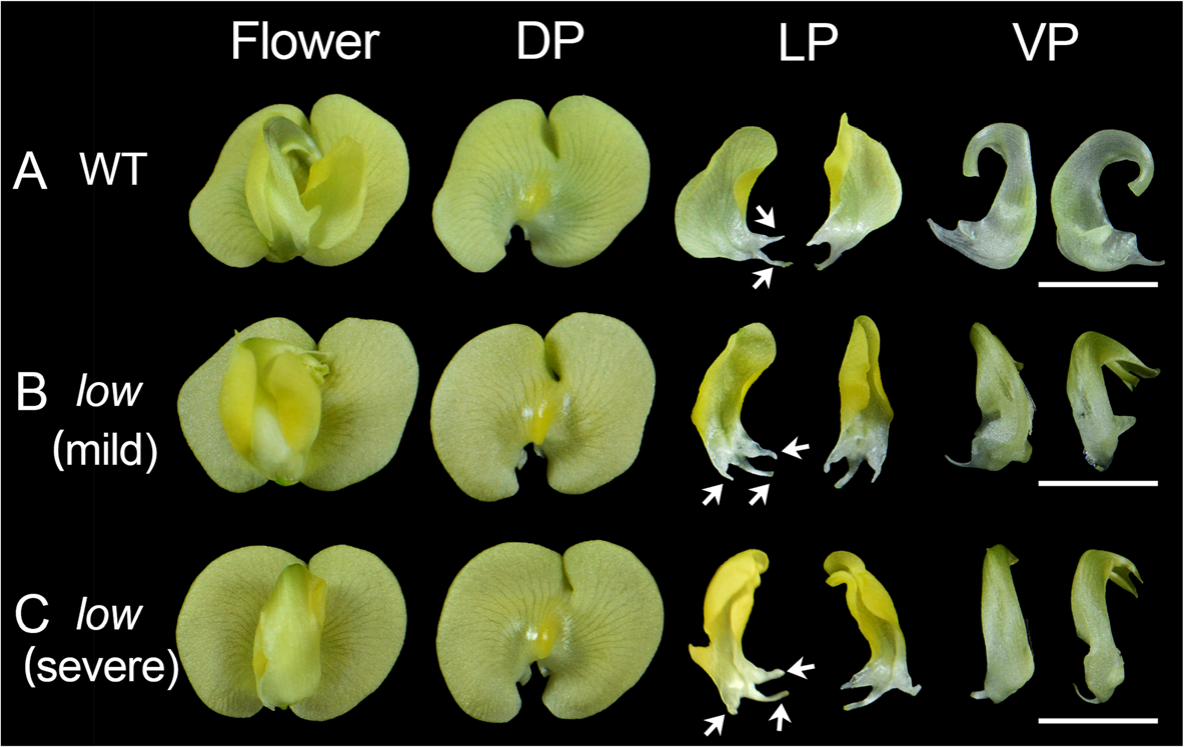
Flowers of in wild-type (WT) and the *love on wings* (*low*) mutant. **a** A WT *Vigna radiata* flower. **b**-**c** Two types of mutant flowers. DP, dorsal petal; LP, lateral petal; VP, ventral petal. The white arrows mark the bulged structures in the base of right later petals. Bars = 10 mm

**Fig. 2:**
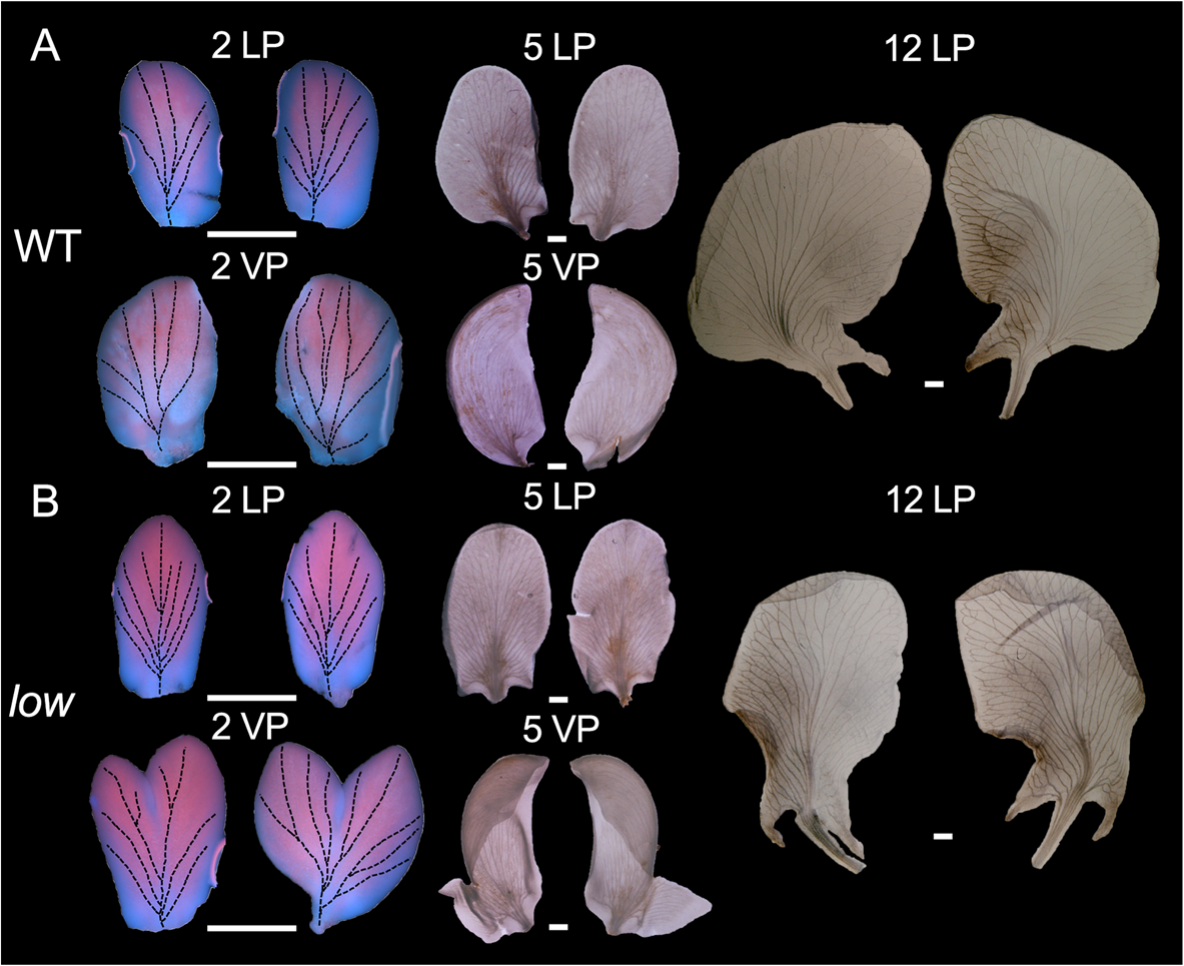
Petal vasculature in wild-type (WT) and mutant. **a** Petals from WT flowers. **b** Petals from mutant flowers. 5 LP, 5 mm lateral petal; 5 VP, 5 mm ventral petal; 12 LP, 12 mm lateral petal. Bars = 2 mm

Using large-scale gamma ray mutagenesis, we characterized one mutant, which showed defects in the landing platform formation (Fig. 1). In the mutant, unlike the WT flower, lateral petals “hugged” ventral petals tightly, thus we named this mutant *love on wings* (*low*). There were basically two different types of flowers on the *low* mutant: the mild type (51 out of 100), exhibited right lateral petal development similar to the WT, but the left lateral petal enclosed the left ventral petal, hindering the formation of the left wing-keel landing platform (Fig. 1b); the other type (49 out of 100) showed severe developmental defects, in which the petal arrangement was so defected since the ventral petal enclosed the lateral petals (Fig. 1c). The two floral morphologies ratio was approximately 1:1. Then, we dissected the newly opened *V. radiata* flower and examined the morphologies of different floral organs. In both types of the mutant flowers, the shapes of lateral and ventral petals had changed (Fig. 1). In the WT flower, two bulged structures grew outwards the base of lateral petals; however, there were three bulged structures in the mutant’s lateral petals (Fig. 1). Both the right and left lateral petals in the mutant became more curved with more symmetrical petal shapes, compared with the WT flower (Fig. 1b-c). Moreover, in the WT flower, two ventral petals formed a keel structure, while in the mutant, a single ventral petal developed into a keel-like shape, similar to the phenotype of a pea mutant, *symmetric petals 1* [7]. We did not find any obvious morphological abnormality in other floral organs. Since the plant organ shape is closely associated with organ vasculature, we then examined the petal vascular pattern in WT and mutant flowers. We dissected the 2 mm and 5 mm flower buds together with 12 mm mature flowers (Fig. 2). We found that in all the developmental stages we examined, lateral petal shape of the WT flower was more asymmetric compared with the mutant lateral petal (Fig. 2). This phenotype is consistent with changes in petal vascular pattern, especially in the main veins (Fig. 2). Petal internal asymmetry and the asymmetric vasculature were further enhanced along with the developmental processes (Fig. 2). As mentioned before, the single ventral petal in the mutant developed into a keel-like structure. This is also evident when we observed the ventral petals from 2 mm or 5 mm flower buds. Additional tissue of ventral petal developed in the 2 mm mutant flower (Fig. 2b). In 5 mm stage, the WT ventral petal exhibited a kidney-like shape and the spur on the left petal has not emerged yet (Fig. 2a). However, additional tissues were further grown on the opposite side of the mutant, forming a keel (Fig. 2b). We also noticed that vascular pattern on the ventral petal was also abnormal compared with the WT (Fig. 2). These results favor the hypothesis that changes in petal shape are linked to the defects in petal vasculature.

### Cloning and phylogenetic analysis of *LOW*

**Fig. 3:**
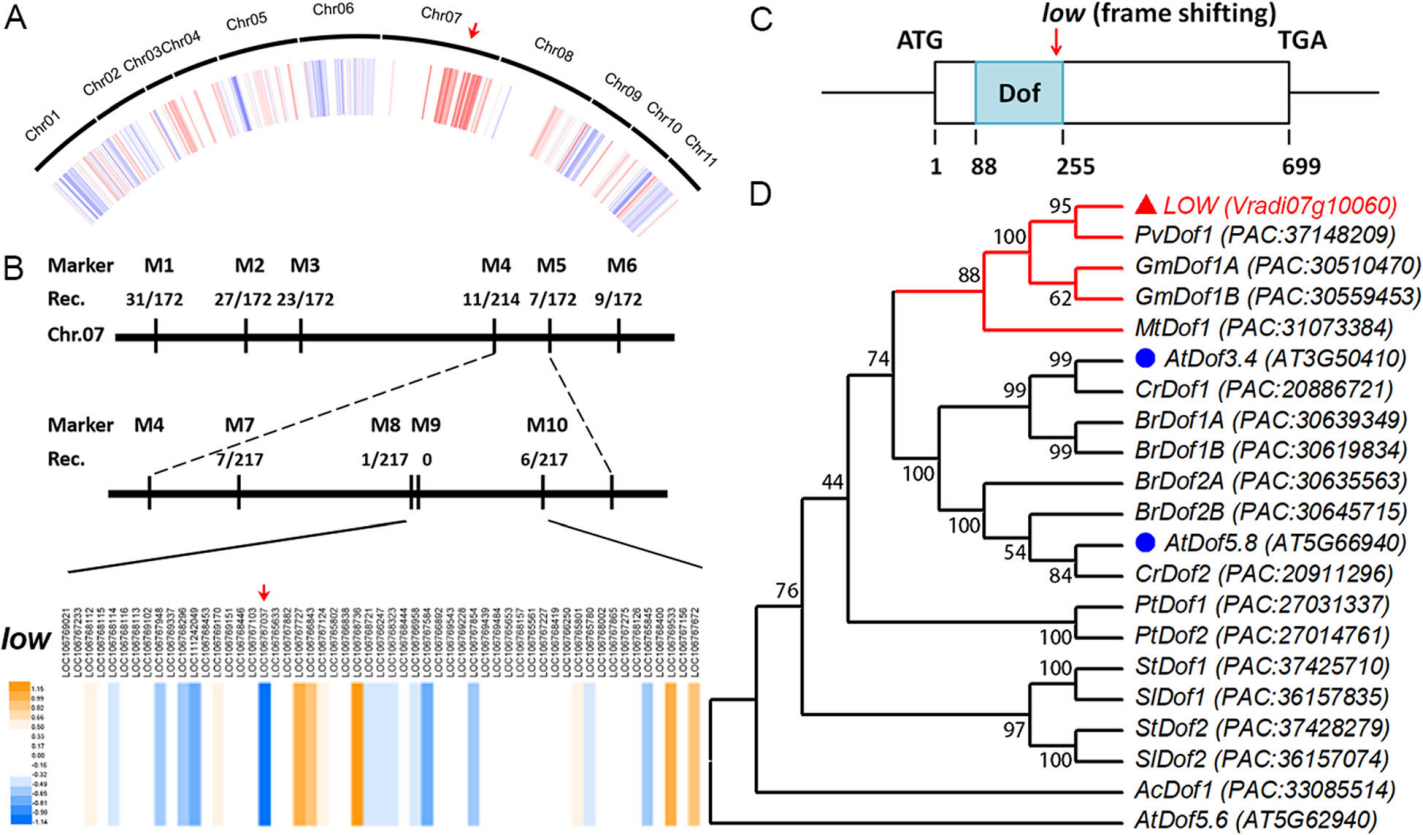
Cloning and phylogeny analysis of *LOW*. **a** Association analysis based on Mutation Mapping Analysis Pipeline for Pooled RNA-seq method. The red region on chromosome 7 indicates strongest association and the red arrow marks the chromosome. **b** Physical map of the large region in Chromosome 7 of *Vigna radiata*, where *LOW* is located. Marker information (M) and recombination frequency (Rec.) are shown. In the lower lane, relative expression heat map of candidate genes between M9 and M10 is shown; the red arrow marks the *Vr07g10060*/*LOC10676703*. **c** The gene structure of *LOW*, nucleotide numbers, start and terminal codons are shown; the red arrow indicates the mutation. **d** Maximum-likelihood tree of *LOW*-like *Dof* genes from *Aquilegia coerulea* (*Ac*), *Arabidopsis thaliana* (*At*), *Brassica rapa* (*Br*), *Capsella rubella* (*Cr*), *Glycine max* (*Gm*), *Medicago truncutula* (*Mt*), *Populus trichocarpa* (*Pt*), *Phaseolus vulgaris* (*Pv*), *Solanum lycopersicum* (*Sl*), *Solanum tuberosum* (*St*) and *Vigna radiata* (*Vr*). 1000 times of bootstrap (value in percentage) is marked at each node and the accession number is presented in the parentheses of each sequence; the red branches highlight *LOW* and its homologs within legume species; the red triangle marks the *LOW* and blue circles indicate two paralogues from Arabidopsis. DOF5.6 was chosen as an outgroup

**Fig. 4:**
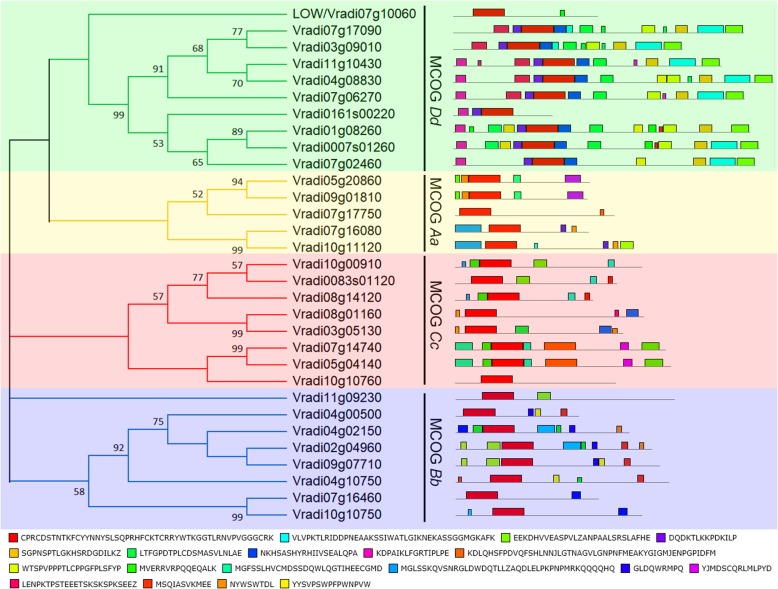
Neighbor-joining tree (left) and motif analysis (right) of 31 Dof proteins from *Vigna radiata* genome. Bootstrapping value is located in each node as percentages (when > 50%) along the branches. Four Major Clusters of Orthologous Genes (MCOG) are shown in different colors. The motif diagrams were generated in MEME, and different colors representing different motifs are shown below

To generate the M2 mapping population, we crossed the original mutant with another cultivar, AL127. Mutation Mapping Analysis Pipeline for Pooled RNA-seq method based on 40 individuals with mutant phenotype suggested that a large region on chromosome 7 would be the possible site where the *LOW* is located (Fig. 3a). *LOW* locus was further mapped and located between two markers, M9 and M10 (Fig. 3b). There are 54 putative genes between them and we found one candidate gene (*Vr07g10060*/*LOC106767037*) significantly down regulated in the mutant (Fig. 3b). *Vr07g10060*/*LOC106767037* encodes a Dof-like transcription factor, and we detected that in the *low* mutant, there was a 2 base-pairs substitution followed by 11 base-pairs deletion in the Dof domain of *Vr07g10060*/*LOC106767037*, leading to a frame-shift and precocious termination of transcription (Fig. 3c and Additional file 2: Figure S2). Subcellular localization assay using Arabidopsis protoplasts demonstrated that green fluorescent protein fused LOW protein was co-localized with a nucleus marker, indicating its function possibly as a transcription factor (Additional file 3: Figure S3). We further analyzed its orthologous proteins in different eudicots lineages (Fig. 3d). In the basal eudicot *Aquilegia coerulea*, only one copy was detected named *AcDof1*. At least one independent duplication event occurred within the diversification of rosids Salicaceae, Brassicaceae and asterids Solanaceae (Fig. 3d). However, in rosids Fabaceae, except for *Glycine max*, in which ancient whole genome duplication once occurred, only one ortholog of *LOW* exists in each legume’s genome (Fig. 3d). To identify the DOF proteins from the mung bean genome, the consensus amino acid sequence of Dof domain was used to BLAST (Basic Local Alignment Search Tool) against its genome database on Legume Information System (https://legumeinfo.org/). Thirty one Dof proteins have been identified and all of them contain a typical Dof DNA binding domain (Additional file 4: Figure S4). To evaluate the evolutionary history among the 31 mung bean Dof proteins, we performed a phylogenetic analysis using their full length protein sequences. Phylogeny tree of these proteins indicated that Dof family have undergone multiple times of duplication (Fig. 4). Based on a previous study [30], the mung bean Dof proteins were divided into four Major Cluster of Orthologous Groups (MCOGs), which could be further divided into multiple subgroups supported by high bootstrap values and motif analysis (Fig. 4). We noticed that although LOW belongs to the MCOG *Dd* group, its sequence is quite different from other MCOG *Dd* members, indicating early divergence of this Dof protein (Fig. 4).

### The spatial-temporal expression pattern of *LOW*

**Fig. 5:**
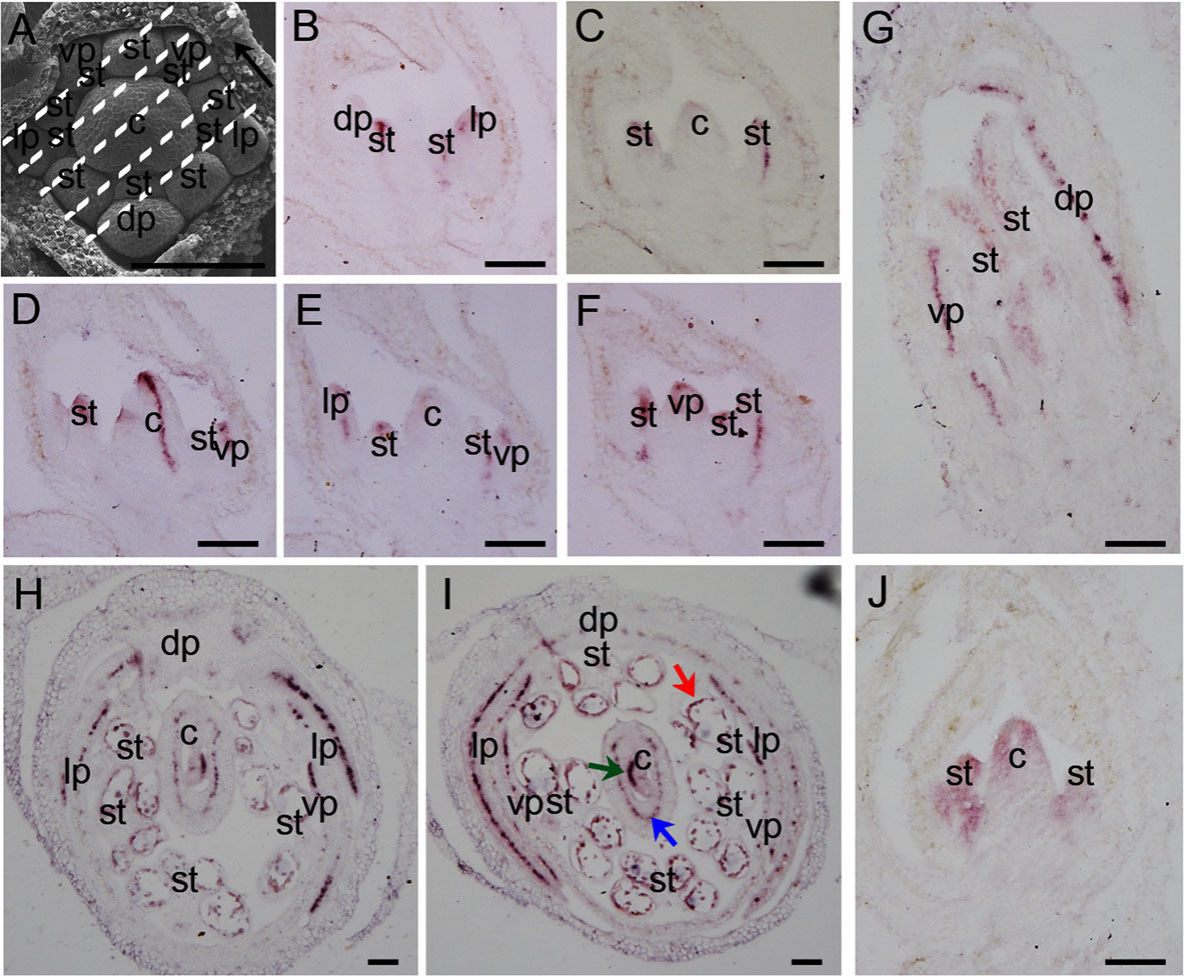
The spatial-temporal expression pattern of *LOW*. **a** Flower organogenesis observed under a scanning electronic microscopy, the dot lines and the black arrow represent the places and direction, where five consecutive longitudinal sections (**b**-**f**) were made; dp, dorsal petal primordium; lp, lateral petal primordium; vp, ventral petal primordium; st, stamen primordium; c, carpel primordium. **b**-**j** The spatial-temporal expression pattern of *LOW* in wild-type (WT) *Vigna radiata* detected by RNA in-situ hybridization. **b**-**j** are longitudinal sections of an early flower bud; **g** shows a longitudinal section of an late flower bud; (**h**-**i**) show transverse sections of a late flower bud; red, green and blue arrows in (**i**) mark the tapetum, ovary and ovule, respectively. Dark brown regions in (**b**-**i**) represent signals detected by *LOW* antisense probe; (J) is a longitudinal section of an early flower bud detected by sense probe of *LOW* as a negative control. Bars = 100 μm

We extracted RNA from various plant tissues, and through qRT-PCR (Quantitative Reverse Transcription Polymerase Chain Reaction), found that *LOW* was highly expressed in the inflorescence with up to 2 mm flower buds (Additional file 5: Figure S5). The expression of *LOW* was rapidly decreased in later flower buds indicating that LOW may function in early flower developmental stages (Additional file 5: Figure S5). We further examined the spatial-temporal expression pattern of *LOW* by RNA in-situ hybridization (Fig. 5a-j). The mRNA of *LOW* accumulated specifically in the central veins of flower organ primordia, including petals, stamens and carpels of early developmental stages (Fig. 5a-f). The longitudinal section of a late stage flower bud showed that *LOW* was expressed in the petals with discontinuously dot-like signals, indicating its expression in secondary petal veins (Fig. 5g). In the transverse sections of a late stage flower bud, the signals of *LOW* were accumulated in defined narrow regions within the petals, which were parallel with the whole flower plane (Fig. 5h-i). Moreover, the mRNA of *LOW* was detected in the anther tapetum, central ovary and ovules within a late flower bud (Fig. 5h-i). A 2 kb DNA fragment of the *LOW* promoter region was fused to a *GUS* (β-glucuronidase) reporter gene (designated as *LOWp:GUS*) and then transformed into Arabidopsis. We detected strong GUS activity in the floral vasculature including pedicels, sepals, petals, filaments, styles and carpels (Additional file 6: Figure S6). The expression pattern of *LOW* in Arabidopsis system is similar to its native expression in *V. radiata*, suggesting that functional analysis of *LOW* in *A. thaliana* may help to understand its roles in *V. radiata*.

### Floral phenotypes of transgenic Arabidopsis

**Fig. 6:**
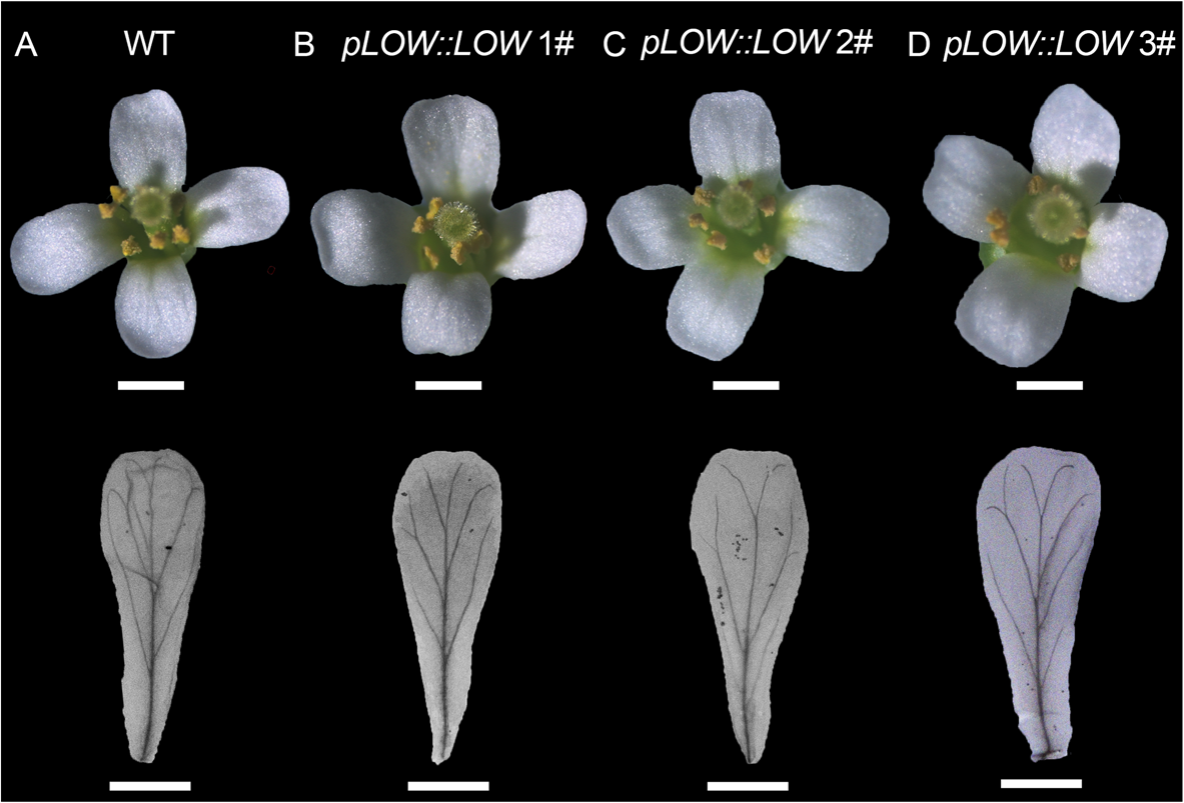
Floral morphology of Col-0 (**a**) and three independent transgenic *Arabidopsis thaliana* lines of *pLOW::LOW* (**b**-**d**). For each plant, the upper panel exhibits an intact flower and the lower panel shows the petal vasculature. Bars =2 mm

Since the 2 kb *LOW* promoter showed specific expression in the floral vasculature of Arabidopsis, we further ectopically expressed *LOW* (designated as *pLOW::LOW*) driven by its own 2 kb promoter. Fifteen independent transgenic lines were obtained, and we carefully examined the floral morphology of each line. Petal shape in the transgenic lines was similar to the wild type plants (Fig. 6a-d). However, when comparing the petal vasculature, we found that in the WT, vascular strands usually formed four enclosed vascular loops emanating from the midvein, while in the *pLOW::LOW* lines, vascular strands failed to form loops (Fig. 6a-d). These results corroborate that LOW functions in floral vasculature pattern.

## Discussion

Co-evolution between plants and their pollinators involves changes of multiple genes among species. Although shift from one pollination syndrome to another requires complex genetic changes, it indeed occurred frequently beyond our expectations. In snapdragon, the ventral petal supported by the lateral petals is incurved at the region named hinge between the petal tube and lobe, forming a landing platform for the insects [31]. A *MIXTA*-like gene *AmMYBML1* reinforces the specialization of ventral petal hinge and thus the formation of the landing platform [31]. In another Lamiales species, *Torenia fournieri*, an ALOG family homolog TfALOG3 is essential for the development of the corolla neck, which may protect their nectar reward to pollinators [32]. In this study, we identified another class of factors from *V. radiata* involved in petal elaboration and keel blossom pattern. Organ asymmetry has been thought to be evolved independently multiple times [33]. In terms of petal, internal asymmetry can be observed either, in dorsal and lateral petals (i.e. snapdragon and wishbone flower), or in lateral and ventral petals (i.e. many keel blossoms). The first factor related to organ asymmetry was characterized in snapdragon. A *CYC*-like gene, *DICHOTOMA*, is expressed in the dorsal half of the dorsal petal primordia [33]. The *cyc dich* double mutant possesses five symmetric ventralized petals, favoring that the CYC-like factors couple floral dorsiventral asymmetry and petal internal asymmetry in *Antirrhinum* [33]. Unlike snapdragon, the ventral petal of a typical keel blossom is asymmetric, thus the ventralized petal should also be asymmetric. This is evident in the *Lotus japonicus CYC* triple mutants, where all petals become asymmetric, indicating that the floral organ internal asymmetry of a keel blossom is also related to CYC-like factors [34]. In pea, we previously isolated several mutants with defects in petal asymmetry, *symmetric petal 1*, *symmetric petal 5*, *elephant ear-like leaf 1* and *bigger organs*. In *syp1–1*, the petals are bilaterally symmetric and increased organs are developed among approximately 1/3 of the flowers, with abnormal primordia initiation found during early developmental stages [7]. Similar to *symmetric petal 1*, mutations in *ELEPHANT EAR-LIKE LEAF 1* and *BIGGER ORGANS* also exhibit several defects in petal asymmetry; these two proteins physically interact with each other and may act in the same genetic pathway [35]. In *symmetric petal 5* and a weaker allele of *bigger organs*, later petals in these mutants become more symmetric compared with the WT’s, and genetic analysis suggests that these two factors act in an addictive manner [35]. Nevertheless, unlike the *low* mutant, these mutants display other pleiotropic phenotypic defects [7, 35, 36]. In the *low* mutant, we only observed the morphological abnormalities in flower perianth, where organ asymmetry in lateral and ventral petals were abolished (Fig. 1). We also found that changes in the asymmetry of vasculature can impact, somehow, the shape of asymmetric petals (Fig. 2), suggesting that petal vasculature development and floral dorsiventral asymmetry may interact with each other, possibly through a direct or indirect regulation of *CYC* genes or other genes involved in floral asymmetry. The transgenic Arabidopsis lines that bare the *LOW*’s promoter and its coding sequence, do not show any obvious changes in petal symmetry (Fig. 6). This could be due to the fact that the *LOW* construct was introduced into a heterologous system (*A. thaliana*) where endogenous *CYC* genes are likely differentially expressed and regulated, compared with what happens in *V. radiata* and other zygomorphic Fabaceae flowers. *LOW* encodes a plant-specific Dof-like transcription factor. Various numbers of *Dof* genes have been found in different plant genomes with different expression patterns [17, 24]. Dof transcription factors play completely distinct roles in plant-specific processes, including light responsiveness, circadian rhythm, seed development, cell cycle regulation, phenylpropanoid metabolism, branching and vascular development [17, 18]. *LOW* was predominantly expressed in the floral vasculature (Fig. 5), which is similar but more specific comparing with the expressions of its orthologs *Dof3.4* and *Dof5.8* in Arabidopsis [23, 26]. According to the phylogenetic tree of *Vigna* Dof proteins, only the MCOG Dd clade that LOW belongs to has strong support, the other clades needs more phylogenetic analyses (Fig. 4). An interesting question is why we observed so specific floral phenotypes in the *low* mutant. Phylogenetic analysis of LOW’s orthologs suggested that this sub-clade of genes has undergone extensively duplication among many other plant lineages including Brassicaceae (Fig. 3d), which might explain the non-redundant function of *LOW* in mung bean. Since the expression of *LOW* is more specific and *pLOW::LOW* transgenic Arabidopsis only shows abnormal vascular pattern rather than shape change, we assume that the role of LOW in vascular patterning is ancient, while its role in petal morphology may be an evolutionary novelty. Dof-like transcription factors work as either transcriptional activators or repressors by binding to the sequences containing the core AAAG motif [18, 37–41]. In *A. thaliana*, a Dof-like transcription factor DOF4.2 negatively affects flavonoid biosynthesis by repressing expression of genes such as *FLAVONOL-3-HYDROXYLASE*, *DIHYDROFLAVONOL REDUCTASE* and *LEUCOANTHOCYANIDIN DIOXYGENASE*, while positively influences the accumulation of hydroxycinnamic acids by promoting the expression of genes including *PHENYLALANINE AMMONIA LYASE*, *CINNAMATE-4-HYDROXYLASE* and *4-COUMAROYL-COA LIGASE 5* [37]. In *Pinus pinaster*, the PpDof5 transcription factor may regulate the expression of glutamine synthetase (GS) genes by activating the transcription of the *GS1b,* or in contrast, by repressing the expression of *GS1a* [38]. In the moss *Physcomitrella patens*, two Dof-like transcription factors, PpDof1 and PpDof2, show transcriptional repressor activities in protoplast transient assays [40]. In the fruit banana *Musa acuminata*, a Dof transcription factor MaDof23 works as a repressor, acting antagonistically in regulating ripening-related genes associated with cell wall degradation and aroma formation [41].

## Conclusions

To summarize, we have characterized a legume *Dof* gene, *LOW*, which is involved in the differentiation of keel blossom by regulating floral vasculature pattern and petal internal asymmetry of mung bean. In the future, it is of interest to study how LOW regulates the petal vasculature and organ asymmetry at a molecular, genetic and developmental level.

## Methods

### Plant materials and map-based cloning

Two cultivars of *V. radiata*, Sulu and AL127, have been purified by selfing for three generations in a greenhouse at 28 ± 2 °C with a 16 h-light/8 h-dark photoperiod at 200 μmol m^− 2^ s^− 1^. *A. thaliana* Col-0 were grown at 20 ± 2 °C with a 16 h-light/8 h-dark photoperiod at 150 μmol m^− 2^ s^− 1^. The seeds of Sulu, AL127 and *A. thaliana* Col-0 were obtained from the germplasm bank in our lab. The gamma ray mutagenesis was performed as we previously described [42]. *low* mutant was isolated from the M2 population of the mutagenized cultivar Sulu background. A 576 F2 mapping population was produced by crossing *low* (from the sulu background) to AL127. RNA-seq libraries based on the published genomic data from 40 individuals with mutant phenotype were generated using Mutation Mapping Analysis Pipeline for Pooled RNA-seq method [43, 44]. This result suggested that a large region on chromosome 7 would be the possible site where the *LOW* mutation is mapped. *Low* was further mapped with the F2 population based on the marker information that we published previously [45]. The primer sequences used in mapping are listed in the Supporting Information (Additional file 7: Table S1).

### Microscopy

Inflorescences or different flower buds were fixed in FAA (3.7% formaldehyde, 50% ethanol, 5% acetic acid) fixative solution prior to clearing in 95% ethanol. Floral organs from buds in a series of developmental stages were dissected and observed under a light or florescence microscope. Petal vasculatures of 5 mm buds and mature flowers became visible under a light microscope after fixation and clearing, while petals from 2 mm buds were observed under the ultra violet laser. For scanning electron microscopy, fixed samples were treated and observed under the Jeol JSM 6360LV (Jeol, Tokyo, Japan) scanning electron microscope as previously reported [46]. Adobe PHOTOSHOP CS6 (Adobe, San Jose, CA, USA) was used to adjust the contrast of the images.

### Phylogeny analysis, motif-based sequence analysis and subcellular localization

For phylogeny analysis of Dof-like family, protein sequences were obtained from the genomic database of *Medicago truncatula* (Mt4.0) and *Vigna radiata* (Vr1.0) in the Legume Information System (https://legumeinfo.org/home), or the Arabidopsis Information Resource (https://www.arabidopsis.org/). Amino acid sequences were aligned using CLUSTALW or MUSCLE followed by the generation of a Neighbor-joining tree with 1000 bootstrap replicates in MEGA6 [47]. Further analysis of LOW sub-clade Dof-like factors, nucleotide sequences from *Aquilegia coerulea*, *Arabidopsis thaliana, Brassica rapa*, *Capsella rubella*, *Glycine max*, *Medicago truncatula*, *Populus trichocarpa*, *Phaseolus vulgaris*, *Solanum lycopersicum*, *Solanum tuberosum* and *Vigna radiata* were obtained from the Phytozome 12 (https://phytozome.jgi.doe.gov/pz/portal.html#). Maximum-likelihood trees of these genes were also generated with 1000 times of bootstrap in MEGA6 [47]. Dof protein sequences were submitted to motif-based sequence analysis website (MEME; http://meme-suite.org/tools/meme) for motif mining under the parameters: -time 18,000, −mod zoops, −nmotifs 50, −minw 6, −maxw 50, −objfun classic, −markov_order 0. For subcellular localization, healthy leaves from 2 to 3 week-old plants of *A. thaliana* were collected for the preparation of protoplasts. The in-frame LOW coding sequence was fused with a green fluorescent protein in the C-terminal region under a constitutive expression promoter *POLYUBIQUITIN 10* and was co-transformed into the leaf mesophyll protoplasts with a nucleus marker, ARF19IV-mCherry, by PEG-induced transformation as previously used [48, 49]. The fluorescent signals were observed using a confocal laser scanning microscopy Zeiss7 DUO NLO (Zeiss, Oberkochen, Germany).

### qRT-PCR and RNA in situ hybridization

Plant genomic DNA and total RNA were extracted from different tissues as described [46]. For qRT-PCR, 1 μg total RNA from different tissues was reverse transcribed using PrimeScript RT reagent Kit with gDNA Eraser (Takara, Beijing, China) following the manufacturer’s instructions. The PCR assays were performed under the manual of LightCycler 480 Real-Time PCR System (Roche, Shanghai, China). Briefly, the target temperature was set to 58 °C and 45 cycles were used for amplification. All the data were normalized against the expression of constitutively expressed reference gene *VrTUB* (*Vradi05g13910*) as reported [50]. The gene expression level was calculated from three biological replicates and three technical replicates. Graphs were produced by GraphPad Prism (GraphPad Software). The primer sequences used in qRT-PCR were listed in the Supporting Information (Additional file 7: Table S1). For RNA in situ hybridization, flowers at different stages of development were fixed and treated as previously reported [51]. DNA Fragment for producing the sense and antisense probes was cloned and ligated to pTA2 plasmid (TOYOBO, Shanghai, China). Probes were then labeled with digoxigenin-UTP (Roche, Shanghai, China). The non-radioactive in situ hybridization processes were carried out as described [52]. The primer sequences used in RNA in situ hybridization were listed in the Supporting Information (Additional file 2: Table S1).

### Arabidopsis transformation and GUS staining

For GUS assay, a 2 kb DNA fragment corresponded to the 5′ promoter and untranslated region of *LOW* was fused to a *GUS* gene on pCXGUS-P vector as described [53]. For functional analysis, the full-length coding sequences of *LOW* was cloned and inserted into pFGC-RCS vector driven by the native 2 kb *LOW* promoter as described [53]. The plasmids were transformed into the EHA105 *Agrobacterium tumefaciens* strains and plant transformation was performed under the instruction of floral dipping method as described [54]. The seeds of transgenic plants were selected on Murashige and Skoog (MS) culture media containing proper antibiotics. Histochemical GUS staining assay was performed as described [55]. The stained tissues were examined, dissected and photographed under a stereomicroscope.

## Supplementary information

**Additional file 1: Figure S1.** Left keel-wing complex in *Vigna radiata* functions as a landing platform for the bee pollinators. (A) Diagram of a *V. radiata* flower, the left keel and left wing are marked in pink and blue, respectively. (B) Front view of a bee visitation. (C) Side view of a bee visitation.

**Additional file 2: Figure S2.** Alignment of *Vr07g10060/LOC106767037* coding sequences in wild type and mutant. Box indicates mutated region in the mutant and the blue line marks the Dof domain.

**Additional file 3: Figure S3.** Subcellular localization of LOW-GFP fused protein. Signals from GFP, mCherry, chloroplast and merged channels are shown; nuclear marker ARF19IV-mCherry plasmid was co-transformed with LOW-GFP construct; Bars = 10 μm.

**Additional file 4: Figure S4.** Dof domain sequence alignment of the 31 mung bean proteins. Identical and similar (> 80%) amino acids are highlighted in black and grey, respectively.

**Additional file 5: Figure S5.** Quantitative RT-PCR analysis of *LOW* in different tissues, error bars of gene expression are ±1 SD from three replicates.

**Additional file 6: Figure S6.** GUS-stained inflorescence and floral organs of transgenic *Arabidopsis thaliana* lines of *LOWp:GUS*. Bar = 2 mm.

**Additional file 7: Table S1.** Primers used in this study.

## Data Availability

The datasets supporting the conclusions of this article are included within the article.

## Abbreviations

BLAST: Basic Local Alignment Search Tool
CYC: CYCLOIDEA
GS: Glutamine synthetase
GUS: β-glucuronidase
LOW: LOVE ON WINGS
MCOGs: Major Cluster of Orthologous Groups
qRT-PCR: Quantitative Reverse Transcription Polymerase Chain Reaction
RNA-seq: RNA-sequencing
WT: Wild-type
