# Supplementary figures and images for: Love on wings, a Dof family protein regulates floral vasculature in Vigna radiata
Source: BMC Plant Biol. 2019 Nov 14;19:495. doi: 10.1186/s12870-019-2099-x (PMC6854777; doi:10.1186/s12870-019-2099-x)

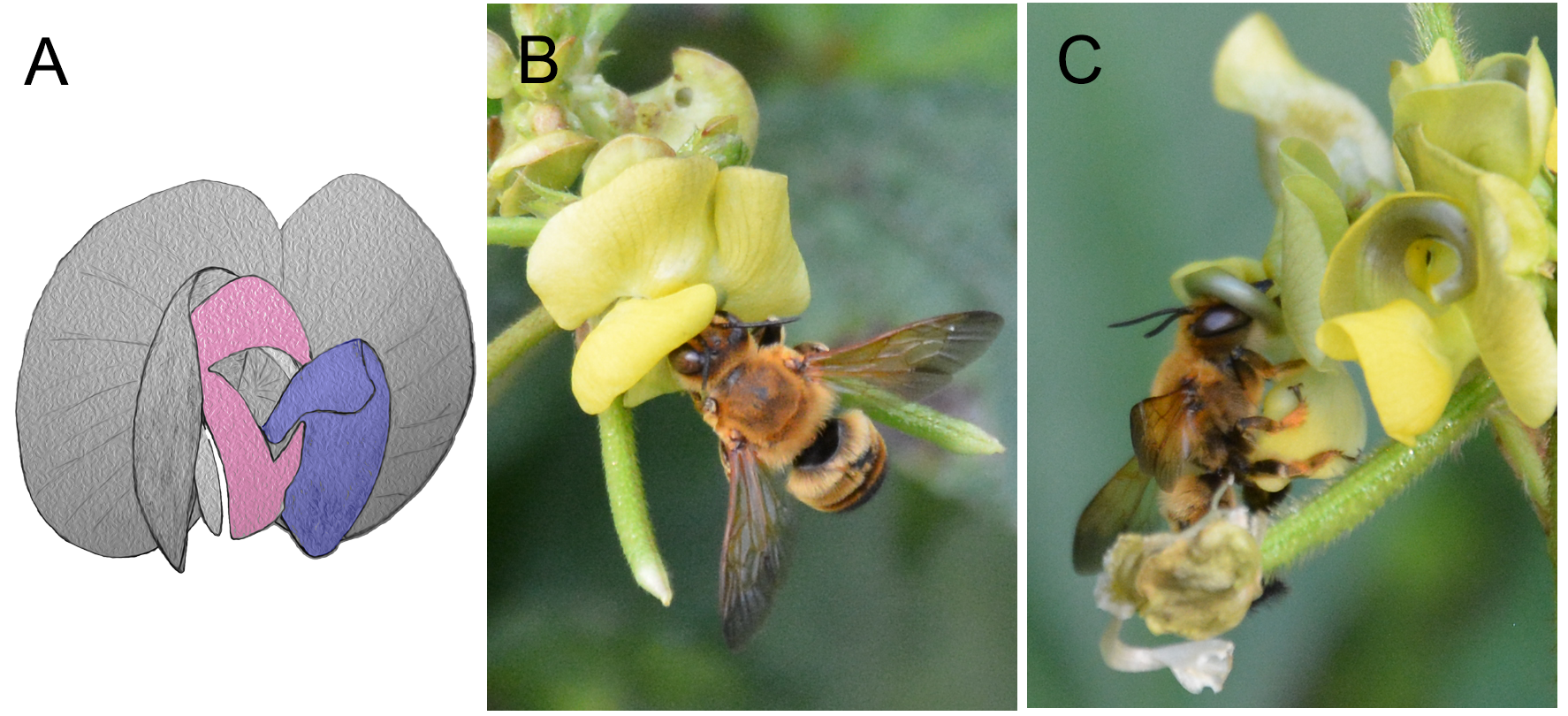

Supplement: Supplementary file 1 — Additional file 1: Figure S1. Left keel-wing complex in Vigna radiata functions as a landing platform for the bee pollinators. (A) Diagram of a V. radiata flower, the left keel and left wing are marked in pink and blue, respectively. (B) Front view of a bee visitation. (C) Side view of a bee visitation. [file 12870_2019_2099_MOESM1_ESM.tif]

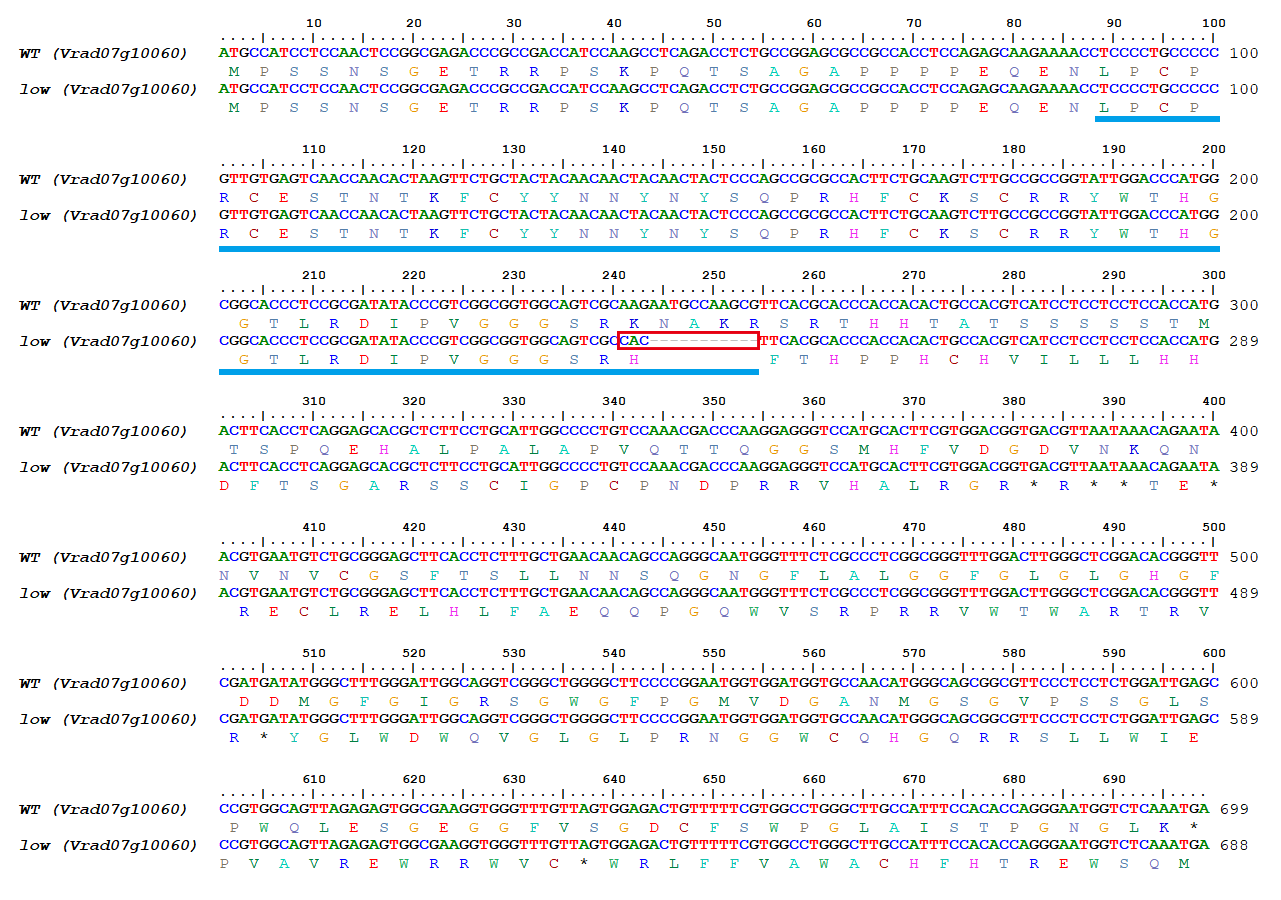

Supplement: Supplementary file 2 — Additional file 2: Figure S2. Alignment of Vr07g10060/LOC106767037 coding sequences in wild type and mutant. Box indicates mutated region in the mutant and the blue line marks the Dof domain. [file 12870_2019_2099_MOESM2_ESM.tif]

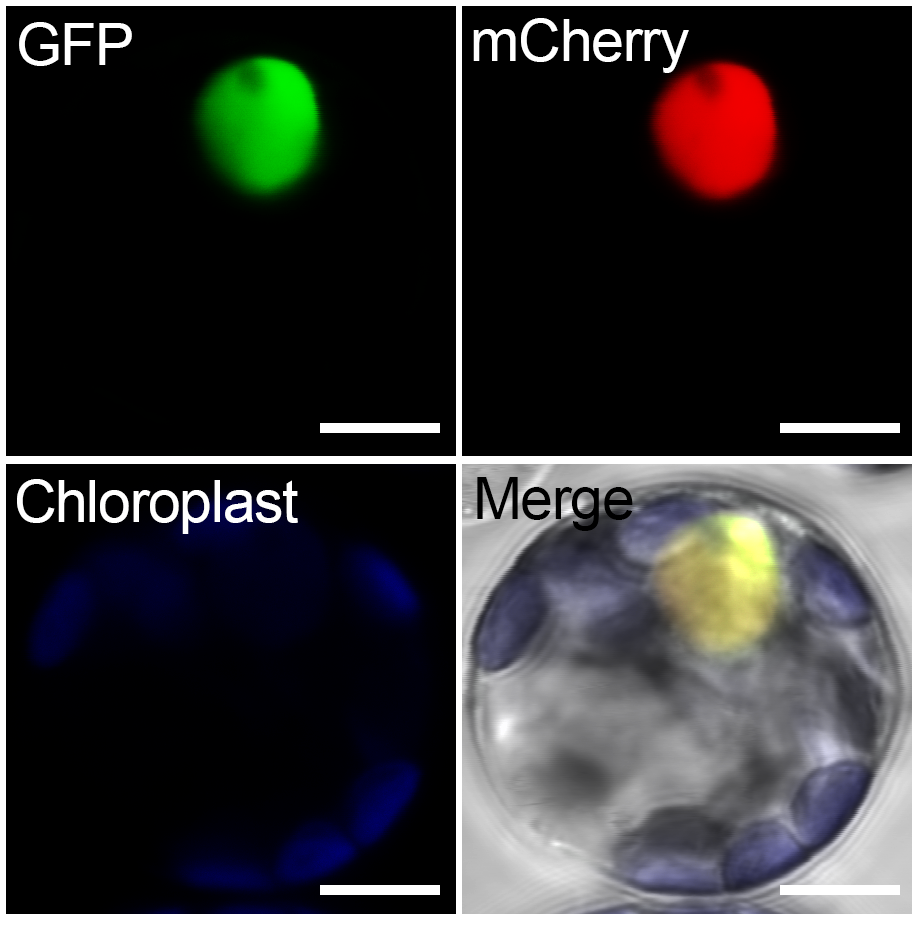

Supplement: Supplementary file 3 — Additional file 3: Figure S3. Subcellular localization of LOW-GFP fused protein. Signals from GFP, mCherry, chloroplast and merged channels are shown; nuclear marker ARF19IV-mCherry plasmid was co-transformed with LOW-GFP construct; Bars = 10 μm. [file 12870_2019_2099_MOESM3_ESM.tif]

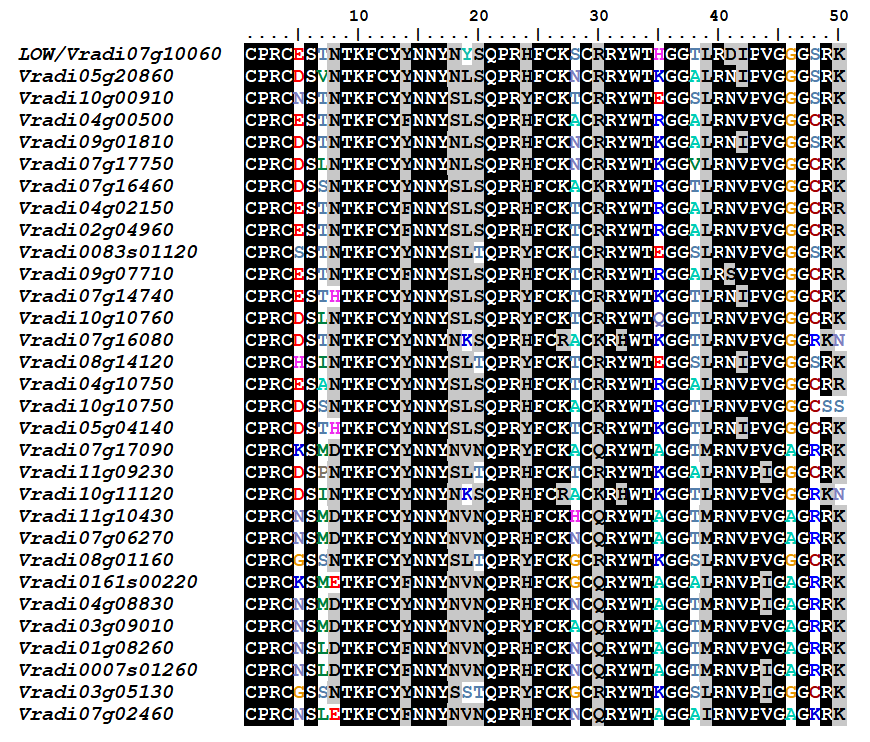

Supplement: Supplementary file 4 — Additional file 4: Figure S4. Dof domain sequence alignment of the 31 mung bean proteins. Identical and similar (> 80%) amino acids are highlighted in black and grey, respectively. [file 12870_2019_2099_MOESM4_ESM.tif]

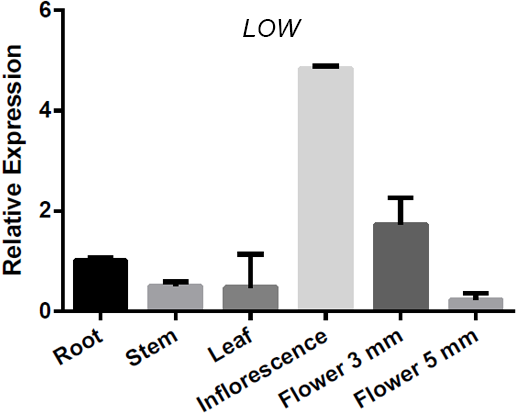

Supplement: Supplementary file 5 — Additional file 5: Figure S5. Quantitative RT-PCR analysis of LOW in different tissues, error bars of gene expression are ±1 SD from three replicates. [file 12870_2019_2099_MOESM5_ESM.tif]

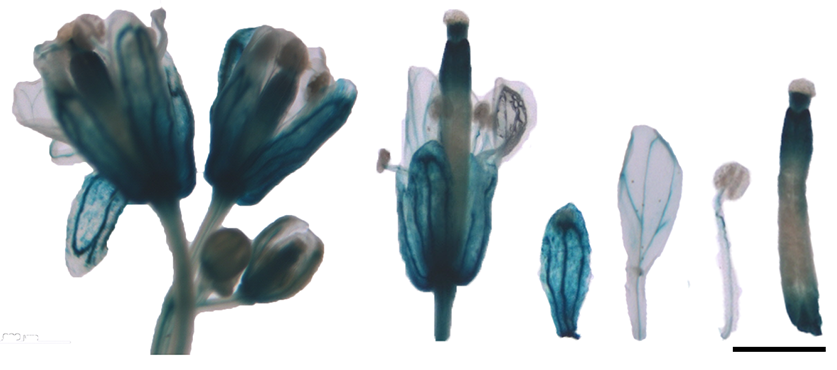

Supplement: Supplementary file 6 — Additional file 6: Figure S6. GUS-stained inflorescence and floral organs of transgenic Arabidopsis thaliana lines of LOWp:GUS. Bar = 2 mm. [file 12870_2019_2099_MOESM6_ESM.tif]
